# The Effect of the Co−Blending Process on the Sensing Characteristics of Conductive Chloroprene Rubber/Natural Rubber Composites

**DOI:** 10.3390/polym14163326

**Published:** 2022-08-16

**Authors:** Zhengming Fan, Rongxin Guo, Zhongyan Yang, Yang Yang, Xingyao Liu

**Affiliations:** 1 Yunnan Key Laboratory of Disaster Reduction in Civil Engineering, Faculty of Civil Engineering and Mechanics, Kunming University of Science and Technology, Kunming 650500, China; 2 Yunnan Sunny Road & Bridge Co., Ltd., Kunming 650500, China

**Keywords:** resistance–strain response, carbon nanotubes, chloroprene rubber/natural rubber composites, conductive networks, interface

## Abstract

Three different blending procedures were used to create multiwalled carbon nanotube (MWCNT)-modified chloroprene rubber (CR)/natural rubber (NR) blended composites (MWCNT/CR–NR). The effects of the blending process on the morphology of the conductive network and interfacial contacts were researched, as well as the resistance–strain response behavior of the composites and the mechanism of composite sensitivity change under different processes. The results show that MWCNT/CR–NR composites have a wide strain range (ε = 300%) and high dynamic resistance–strain response repeatability. Different blending procedures have different effects on the morphology of the conductive network and the interfacial interactions of the composites. If the blending procedures have wider conductive phase spacing and stronger interfacial contacts, the change in the conductive path and tunneling distance occurs more rapidly, and the material has a higher resistance–strain response sensitivity.

## 1. Introduction

Conductive polymer composites (CPCs) are of interest due to their exceptional fatigue resistance, great strain–sensing capabilities, outstanding flexibility, and large deformation capabilities [1,2]. In recent years, CPCs have been studied extensively as strain–sensing materials in biomedical [3], flexible electronic skin [1], human motion detection [4], and structural health monitoring [5] applications. It is possible to combine the good qualities and synergistic effects of CPCs by doping them with nanofillers in a polymer matrix. As such, the choice of conductive nanofillers and matrix materials has a significant impact on the composites’ performance [6].

Carbon nanotubes (CNTs) have many advantages, including low density, high strength, a large aspect ratio, excellent electrical conductivity, and outstanding mechanical properties [7]. Various CNTs, particularly multiwalled carbon nanotubes (MWCNTs), offer much potential as polymer–reinforcing materials [8]. Rubber is extensively utilized as a matrix material for CPCs because of its excellent mechanical properties, especially its high elasticity and resistance to damage. Natural rubber (NR) provides numerous benefits in strain−sensing matrix materials, including high viscoelasticity, large deformation capacity, plasticity, and water resistance [9]. However, NR is not a good substrate for sensing materials because of its poor aging resistance, poor high temperature resistance, and low tensile strength [10], which affects its service life and long-term monitoring efficacy in complex service environments. Chloroprene rubber (CR) is a high-strength rubber with exceptional weathering, heat, and age-resistance properties [11]. Blending CR and NR can combine the benefits of CR and NR while also improving NR’s aging resistance, which is critical for expanding NR’s application range [12].

The resistance–strain response behavior of CPCs is key to their use as reliable strain-sensing materials. Conductive network morphology and interfacial interactions play an important role in influencing the sensing behavior of CPCs [13]. Currently, researchers have proposed many methods to control the conductive network morphology and interfaces in CPCs. Lin and Alig et al. [14,15] used shear methods (e.g., stretching, injection molding, and prestrain) to distribute the conductive fillers directionally. Jung et al. [16] used thermal annealing above the melting or glass transition temperatures of the polymer matrix to repair conductive contacts between local conductive regions. George et al. [17] discovered that emulsion grade mixing of MWCNTs and NR resulted in the formation of segregated conductive networks. Although the methods described above may efficiently manage the conductive network topology and interfacial interactions in CPCs, the resistance–strain response characteristics have received little attention [13]. Polymer blending can build conductive networks with selective distribution to control the resistance–based strain-sensing behavior [18,19,20], and the process serves as a new way to prepare high-performance CPC strain−sensing materials with tunable sensing behavior [21]. However, most of the polymer blends currently tested are used in a molten state [22,23]. Investigating the conductive network morphology and interfacial interactions under various blending processes, as well as their relationship with strain–sensing behavior, is critical for promoting the development of polymer blends in the field of strain sensing and providing a reference for the selection of superior blending processes.

In this work, three different blending procedures were employed to manufacture MWCNT-modified CR/NR-based composites using MWCNTs as a conductive filler and CR/NR blends as a matrix. The dispersion state of MWCNTs in the CR/NR blended matrix was investigated, as well as the interfacial interaction between the composite matrix and filler under different preparation processes. The aims of these investigations were to reveal the effects of the conductive filler’s dispersion state and to elucidate the interfacial interaction between the filler matrix and the resistance–strain response behavior and to explain the material resistance–strain response mechanism based on the tunneling effect.

## 2. Materials and Methods

### 2.1. Raw Materials

**Figure 1:**
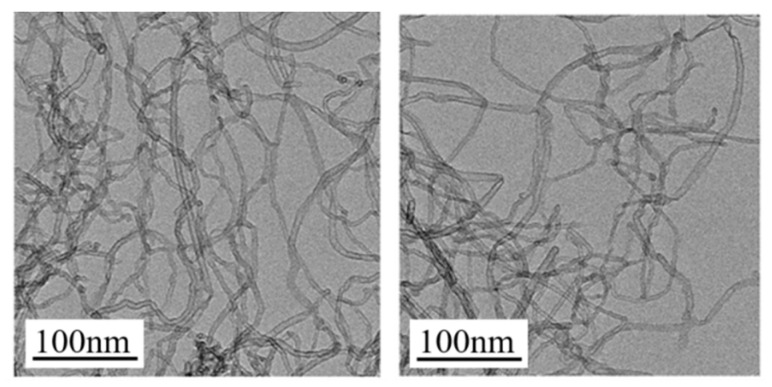
The diameter and micromorphology of the original multiwalled carbon nanotubes were characterized by transmission electron microscopy. (Note: the data used in this figure are provided by the Chengdu Organic Chemicals Co., Ltd., Chinese Academy of Sciences, Chengdu, China).

Chloroprene rubber latex (CRL, grade SNL511A, solid content 50 wt.%, Jinan Deqiao Chemical Technology Co., Ltd., Jinan City, Shangdong, China), natural rubber latex (NRL, solid content 60 wt.%, Guangdong Maoming Zhengmao Petrochemical Co., Ltd., Maoming City, Guangdong, China) and MWCNTs (length 10–20 μm, OD 4–6 nm, as shown in Figure 1, specific surface area 500–700 m^2^ g^−1^, purity > 98%, China Organic Chemical Co., Ltd., Chinese Academy of Sciences, Chengdu, China) were used. The remaining reagents were commercially available and were not further processed, including sodium dodecyl sulfate, magnesium oxide, zinc oxide, sulfur, stearic acid, *N*-isopropyl-*N*′-phenyl-*p*-phenylenediamine, and *N*-*tert*-butyl-2-benzothiazole hyper-sulfonamide.

### 2.2. Preparation of MWCNT/CR–NR Composites

In this study, MWCNT/CR–NR composites were prepared using a four-step emulsion blending method according to the mixing proportions described in Table 1.

**Figure 2:**
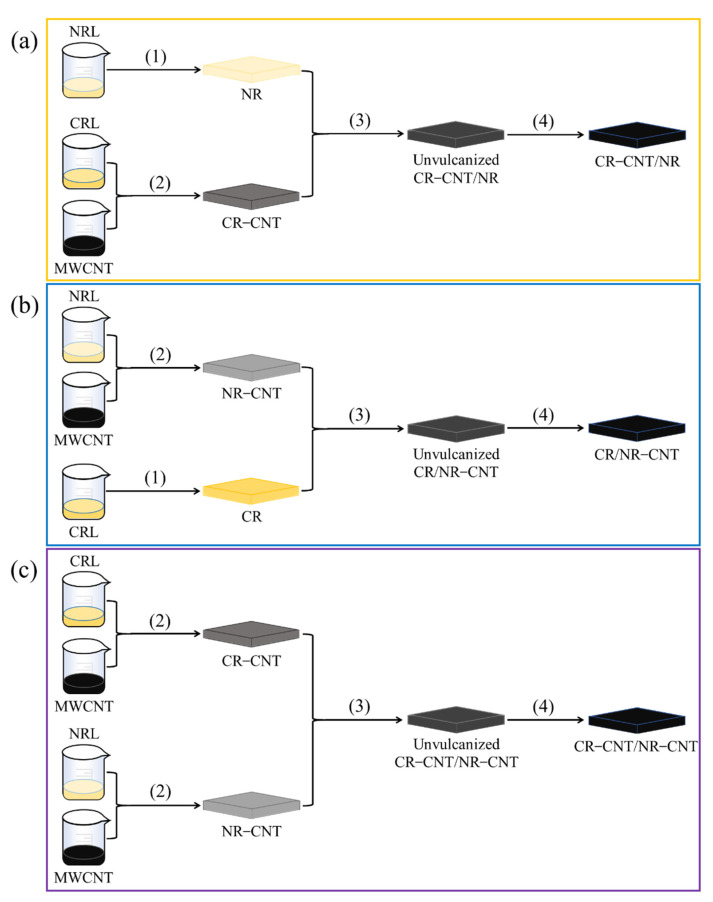
A schematic diagram of the three co-blending processes: (**a**) CR–CNT/NR, (**b**) CR/NR–CNT, and (**c**) CR–CNT/NR–CNT composites.

(1) Preparation of pure rubber: To obtain the flocculated colloid, the latex was weighed in a beaker, calcium chloride flocculant with a mass fraction of 0.01 was added gently to the latex, and the latex was rinsed repeatedly with deionized water when flocculation was complete. Then, the sample was passed through a two-roller refiner (the speed ratio of the rotor was 1:1.2, the same below) for 15 min to obtain pure CR and pure NR.
(2) Preparation of MWCNT/rubber blends: The MWCNTs were weighed in a beaker, a small amount of deionized water was added and ultrasonically dispersed for 10 min to obtain a stable MWCNT dispersion. Then, the latex was poured into the MWCNT dispersion and mixed with sufficient stirring to obtain the MWCNT/latex mixture. Calcium chloride flocculant with a mass fraction of 0.01 was slowly added to the mixture to obtain MWCNT/rubber flocculant, which was repeatedly rinsed with deionized water to obtain MWCNT/rubber flocculant colloid. Finally, the MWCNT/CR blends and MWCNT/NR blends were produced by passing the samples through a two-roller kneading machine for 15 min.
(3) Rubber blending: The MWCNT/CR–NR blends were prepared by three blending methods (as shown in Figure 2): (3–1) MWCNT/CR blends were blended with pure NR in a kneader for 2 min and then mixed with other vulcanizing reagents for 4 min (as shown in Figure 2a); (3–2) MWCNT/NR blends were blended with pure CR in the kneader for 2 min and then mixed with other vulcanizing reagents for 4 min (shown in Figure 2b); (3–3) MWCNT/CR compounds were mixed with MWCNT/NR compounds in the kneader for 2 min and then mixed with other vulcanizing reagents for 4 min (shown in Figure 2c).
(4) Vulcanization: The MWCNT/CR–NR compound was vulcanized at 150 °C and 10 MPa for 10 min to obtain the MWCNT/CR–NR composite.

In this paper, samples are denoted as A–xCNT/B y/z, where A and B denote the rubber matrix, x denotes the amount of MWCNTs in A rubber, and y and z denote the number of parts of A rubber and B rubber, respectively. For example, CR–6CNT/NR 5/5 indicates that the MWCNTs were first premixed in the CR phase, and then, CR–MWCNTs containing 6 wt.% MWCNTs were mixed with NR containing 5 parts of CR and 5 parts of NR.

### 2.3. Methods

#### 2.3.1. Measurement of Contact Angle

DataPhysics OCA 20 was used to measure contact angles. For contact angle measurements, pure CR and NR samples were compression molded at 150 °C under 10 MPa pressure for 10 min. The contact angle measurements were also used to determine the surface tension, dispersion, and polar components of CR and NR.

#### 2.3.2. Mechanical Properties

According to ISO 37:2005(E), the mechanical properties of dumbbell standard specimens of CR/NR blends and MWCNT/CR–NR composites were measured using a tensile and compressive test system (DDL–10, Changchun Institute of Testing Machines, Changchun, China) with a maximum load of 10 kN controlled by displacement at ambient temperature and a loading rate of 200 ± 5 mm/min. Each experiment had a minimum of three samples.

#### 2.3.3. Resistance–Strain Response Behavior

The resistance–strain response properties of MWCNT/CR–NR composites were measured using electronic universal testing equipment with a test length of 20 mm. A 34410A digital multimeter (Keysight Technologies, Inc., Santa Rosa, CA, USA) was used to measure resistance throughout the loading process [7].

#### 2.3.4. FE–SEM

At a 10 kV accelerated voltage, FE–SEM (NOVA Nano SEM450, FEI Company, Hillsboro, OR, USA) was employed to analyze the morphology of the MWCNT/CR–NR composites. Samples were frozen in liquid nitrogen for 10 min before being broken with an external pressure source to generate fresh fracture surfaces. To increase the conductivity and generate better FE–SEM images, the fracture surface of the sample was sprayed with platinum.

#### 2.3.5. Raman Spectroscopy

The contact was analyzed using Raman spectroscopy (HORIBA Scientific LabRAM HR Evolution, HORIBA Jobin Yvon, Paris, France) with a Raman shift of 800–2000 cm^−1^.

#### 2.3.6. Conductivity

In order to measure the electrical conductivity of MWCNT/CR–NR composites, a 34410A digital multimeter was used to test the electrical properties of composites (sample size: 40 mm × 10 mm × 1 mm). The test uses the two-wire method.

The conductivity formula of the MWCNT/CR–NR composite is as follows:

(1) $$\sigma =\frac{1}{\rho }=\frac{L}{RS}$$

where *σ* is the conductivity, *ρ* is the resistivity, *L* is the length of the sample, *R* is the resistance of the sample, and *S* is the cross–sectional area of the sample.

## 3. Results

### 3.1. Mechanical Properties of CR/NR Blended Rubber

**Figure 3:**
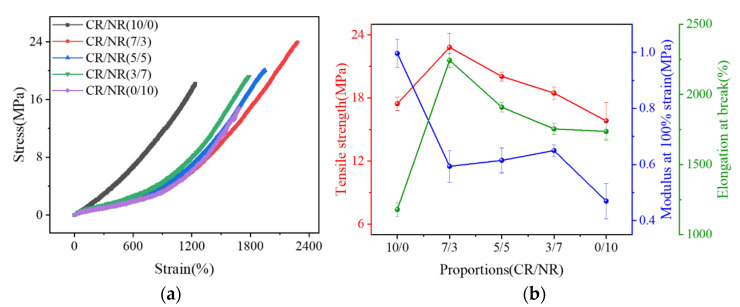
Stress–strain curves of CR/NR blend with different blending ratios (**a**) and their tensile strength, modulus, and elongation at pull−off (**b**).

The stress–strain curves of CR/NR blends with various blending ratios are reported in Figure 3a. NR (CR/NR = 0/10) displayed a much larger strain than CR (CR/NR = 10/0) at the same stress level. Pure CR showed a stronger tensile strength and modulus at 100% strain than pure NR but a lower elongation at pull-off, as demonstrated in Figure 3b. These results indicate that the stiffness of CR is greater than that of NR. As CR was blended with NR, the tensile strength and elongation at pull-off of the CR/NR blends increased compared to their single components, and these properties increased as the CR/NR blending ratio rose. Furthermore, the modulus of CR/NR blends was between that of pure CR and pure NR, and it decreased as the CR/NR blending ratio increased. These results demonstrate that blending CR and NR may fully combine their individual advantages and result in a blended rubber with better mechanical properties than each on their own.

### 3.2. Mechanical Properties of MWCNT/CR–NR Composites

**Figure 4:**
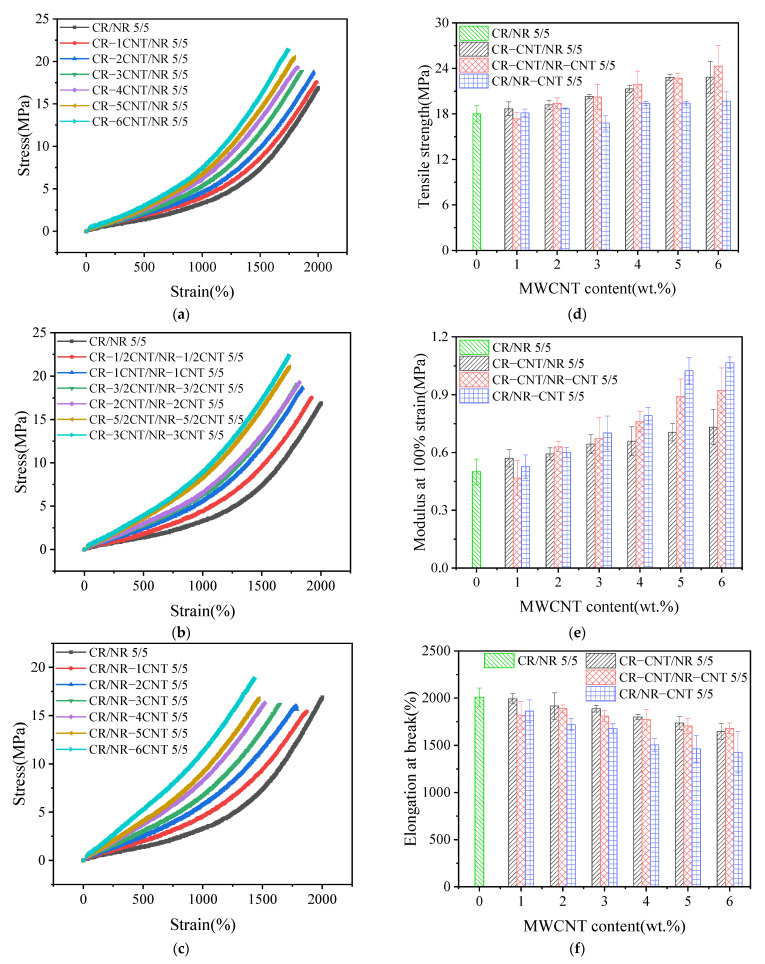
The mechanical properties of MWCNT/CR−NR composites with different blending process: the stress–strain curves ((**a**) CR−CNT/NR composites, (**b**) CR−CNT/NR−CNT composites, and (**c**) CR/NR−CNT composites), (**d**) tensile strength, (**e**) modulus at 100% strain, and (**f**) elongation at break.

Figure 4 presents the test results of various mechanical properties of MWCNT/CR–NR composites under different blending processes. Figure 4a–c show the stress–strain curves of composites with different MWCNT content under three blending processes. It can be clearly seen that stress increases with the increase in strain and MWCNT content no matter which blending process is used. This indicates that the stiffness of the material increases with the increase in MWCNT content. At the same time, composites with different blending processes show an excellent stress–strain relationship. As shown in Figure 4d, the tensile strength of composites with different blending processes also increases with the increase in MWCNT content. When the MWCNT content was 6 wt.%, the tensile strengths of CR–6CNT/NR, CR–3CNT/NR–3CNT, and CR/NR–CNT composites were increased to 22.8 MPa, 24.3 MPa, and 19.7 MPa, respectively, which were 26.7%, 35.0%, and 9.4% higher than that of CR/NR blends (18.0 MPa). The main reason is that the incorporation of MWCNTs restricts the movement of rubber molecular chains and increases the stiffness and strength of the material [24]. Compared with the other two blending methods, CR–CNT/NR–CNT composites have higher tensile strength, which is related to the morphology of the conductive network formed by different blending methods. The modulus at 100% strain of composites with different blending processes has a similar trend to the tensile strength, as shown in Figure 4e. When the MWCNT content increased to 6 wt.%, the moduli of CR–6CNT/NR, CR–3CNT/NR–3CNT, and CR/NR–6CNT composites were increased to 0.7 MPa, 0.9 MPa, and 1.1 MPa, respectively. While the elongation at break decreased gradually with the increase in MWCNT content, as shown in Figure 4f. When the MWCNT content increased to 6 wt.%, the elongation at break of CR–6CNT/NR, CR–3CNT/NR–3CNT, and CR/NR–6CNT composites decreased from 2004.8% to 1648.1%, 1681.4%, and 1426.4%, respectively. The change in modulus and elongation at break is due to the increase in MWCNT content, which means more contact surface between MWCNTs and rubber matrix, leading to stronger physical entanglement and interface interaction, and resulting in increased stiffness of the composites [25].

### 3.3. Conductivity

**Figure 5:**
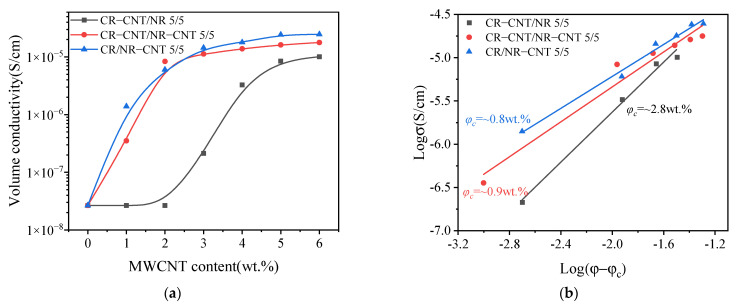
(**a**) The relationship between volume conductivity and MWCNT content of composites with different blending processes; (**b**) fitting curve of percolation threshold.

The conductivity of composites depends on their dispersion and conductive network structure [26]. Figure 5 shows the curved relationship between MWCNT content and the conductivity of composites under different blending processes. Due to the limited range of the instrument, only the conductivity of composites with conductivity greater than 2.67 × 10^−8^ S cm^−1^ was tested. As shown in Figure 5a, the conductivity of the composites with the three blending processes increases with the increase in MWCNT content. When the content of MWCNT increases to a certain range, the conductivity of the material rises sharply and percolation conduction behavior appears [27], because, at this time, MWCNTs have formed a conductive network in the matrix to ensure that the materials have conductive ability. According to tunnel effect theory [28], the relationship between the conductivity of composites and conductive fillers is shown as follows:

(2) $$\sigma =\sigma_{0}{\left(\varphi -\varphi_{c}\right)}^{\xi },\;\varphi >\varphi_{c}$$

Take the logarithm of the left and right sides of Equation (2):

(3) $$\mathrm{Log}\sigma =\mathrm{Log}\sigma_{0}+\xi \mathrm{Log}(\varphi -\varphi_{c})$$

where *σ* is the volume conductivity of the composites, *σ*_0_ is the scaling factor (constant varying with the filler and matrix), *φ* is the content of MWCNTs, *φ_c_* is the percolation threshold, and *ξ* reflects the dimension of MWCNT conduction network.

According to Equation (3), draw the *Logσ − Log(φ − φ_c_)* scatter diagram and fit it with the least squares method, as shown in Figure 5b. The percolation thresholds of CR–CNT/NR, CR–CNT/NR–CNT, and CR/NR–CNT composites are 2.8 wt.%, 0.9 wt.%, and 0.8 wt.%, respectively. The different conductive properties of CR–CNT/NR, CR–CNT/NR–CNT, and CR/NR–CNT composites are related to the different conductive network morphology formed by the three blending processes, which will be explained in detail later.

### 3.4. Resistance–Strain Response Characteristics of MWCNT/CR–NR Composites

#### 3.4.1. Resistance–Strain Response Behavior under Uniaxial Strain

**Figure 6:**
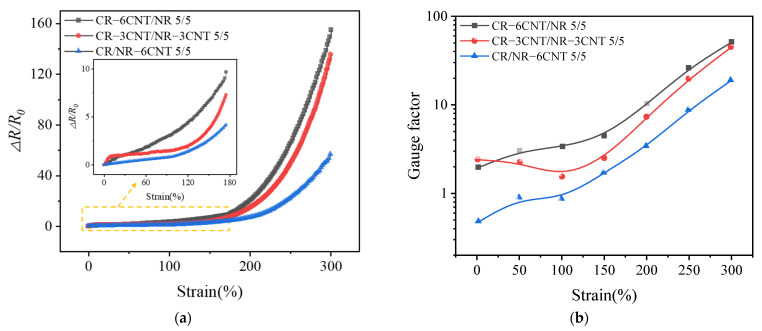
The relationship between ∆*R*/*R*_0_ (**a**) and gauge factor (**b**) and applied strain for composite materials.

To assess the potential of MWCNT/CR–NR composites as strain sensors, the resistance–strain response behavior of MWCNT/CR–NR composites was investigated using the relationship between ∆*R*/*R*_0_ (∆*R* = *R* − *R*_0_, where *R* and *R*_0_ denote the applied strain *ε* and the initial strain *ε*_0_ at the time of resistance, respectively) and *ε*, where the strain rate is 100 mm/min. As illustrated in Figure 6a, the value of ∆*R*/*R*_0_ grew as *ε* increased. This increase in the ∆*R*/*R*_0_ value was due to the weakening of the electron tunneling effect, which was caused by the breakage of the MWCNT network and the increase in the distance between MWCNTs during uniaxial strain, both of which cause an increase in resistance [29]. Furthermore, at the same strain level, the ∆*R*/*R*_0_ values of the CR–3CNT/NR–3CNT composites were greater than those of CR/NR–6CNT, while the ∆*R*/*R*_0_ values of the CR–6CNT/NR composites were higher than those of CR–3CNT/NR–3CNT. A gauge factor (GF) was proposed to quantitatively measure the sensitivity of the resistance–strain response of the composite: GF = (∆*R*/*R*_0_)/*ε*. The sensitivity increased as the GF value increased. As shown in Figure 6b, the magnitudes of the sensitivities of the composites produced by the three blending processes remained consistent with their ∆*R*/*R*_0_ values. Because the varied blending techniques resulted in distinct initial conductive network geometries, these composites had different sensitivities [30]. Among them, the CR–3CNT/NR–3CNT composites showed a decrease in sensitivity in the strain range of 0–100%. This is due to the uniform and dense distribution of conductive fillers in the CR–3CNT/NR–3CNT composites, forming a more stable conductive network. Under the initial tensile strain, the conductive network is damaged to a small extent. Therefore, the resistance of the composites does not change significantly compared to the strain, so the sensitivity decreases [31]. The MWCNT/CR–NR composites had a 300% strain range, indicating their potential application in large strain monitoring.

#### 3.4.2. Resistance–Strain Response Behavior under Cyclic Loading

**Figure 7:**
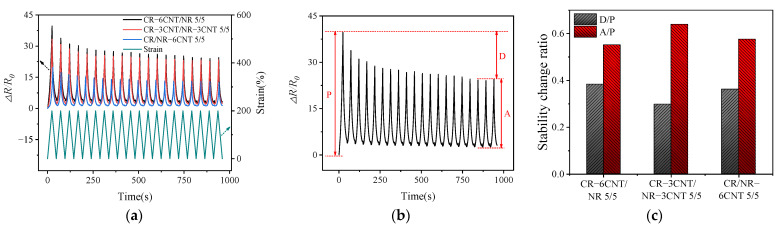
(**a**) The variation of ∆*R/R*_0_ under cyclic strain in MWCNT/CR−NR composites, (**b**) schematic diagram of P, D, A, (**c**) stability of resistance variation under different co−blending processes.

The sensitive and stable dynamic resistance–strain response behaviors of CPCs are essential for their application as strain response materials. The resistance–strain response behavior of MWCNT/CR–NR composites made by three different blending processes was investigated under cyclic strain, with a strain rate of 100 mm/min and a strain amplitude of 200%. As shown in Figure 7a, the ∆*R*/*R*_0_ amplitude progressively declined at the start of cyclic loading, and the reduction in ∆*R*/*R*_0_ amplitude dramatically reduced with the increase in the number of cycles and eventually stabilized. This behavior is due to the rupture of the interface between the MWCNTs and the matrix during the first few cycling stages that formed new conductive paths [32], which resulted in a decrease in the resistance of the composite. After several cycles, the competition between the breakdown and formation of the conductive network was gradually balanced, and the resistance eventually stabilized [33]. Under cyclic loading, the MWCNT/CR–NR composites possessed high repeatability of the resistance–strain response and strain monitoring potential. “Shoulder peaks” were also detected in CR–6CNT/NR, CR–3CNT/NR–3CNT, and CR/NR–6CNT composites, as described in many publications. The competition between the breakdown and repair of the conductive network under cyclic stress, as well as the viscoelasticity of the substrate, are the primary causes of the “shoulder” phenomena [33]. Furthermore, by comparing the magnitude of the ∆*R*/*R*_0_ values of the composites with different blending processes, the CR–6CNT/NR composite had the greatest amplitude of resistance change, indicating that it had the greatest resistance–strain response sensitivity, which was consistent with the phenomenon observed in Figure 6.

The stability of ∆*R*/*R*_0_ amplitude variation in MWCNT/CR−NR composites was further investigated utilizing *D*/*P* and *A*/*P* values (the *P*, *D*, and *A* schematics are given in Figure 7b) to assess the stability of ∆*R*/*R*_0_ variation under different co-blending processes, as shown in Figure 7c. By comparing the *D*/*P* and *A*/*P* values of the three blending process composites, it was found that the CR–3CNT/NR–3CNT composites had smaller *D*/*P* values and larger *A*/*P* values, indicating a better stability of the ∆*R*/*R*_0_ amplitude change. This stability is closely related to the good dispersion of the conductive filler in the matrix.

## 4. Discussion

### 4.1. Interaction between Filler and Matrix Interface

The interfacial interaction of MWCNTs and polymers is a critical aspect in obtaining high-performance MWCNT/polymer composites. To explore the effects of different blending processes on the interfacial interactions of MWCNT/CR–NR composites, the thermodynamic work of adhesion was calculated, and Raman analysis was performed.

The magnitude of the adhesion between MWCNTs and the rubber matrix in different blending process composites can be characterized by the thermodynamic work of adhesion ($W_{AB}$) [21].

(4) $$W_{AB}=2\sqrt{\gamma_{A}^{d}\gamma_{B}^{d}}+2\sqrt{\gamma_{A}^{p}\gamma_{B}^{p}}$$

where $W_{AB}$ is the thermodynamic work of adhesion between components *A* and *B*. $\gamma_{A}$ and $\gamma_{B}$ are the surface tensions of components *A* and *B*, respectively. $\gamma_{A}^{d}$ and $\gamma_{B}^{d}$ are the dispersion values of the surface tensions of components *A* and *B*, respectively. $\gamma_{A}^{p}$ and $\gamma_{B}^{p}$ are the polarity values of the surface tensions of components *A* and *B*, respectively.

The surface tension, dispersion and polarity values can be calculated from the contact angle data by Equations (5) and (6) [34].

(5) $$\left(1+\cos\theta_{H_{2}O}\right)\gamma_{H_{2}O}=4\left(\frac{\gamma_{H_{2}O}^{d}\gamma^{d}}{\gamma_{H_{2}O}^{d}+\gamma^{d}}+\frac{\gamma_{H_{2}O}^{p}\gamma^{p}}{\gamma_{H_{2}O}^{p}+\gamma^{p}}\right)$$

(6) $$\left(1+\cos\theta_{{\left(CH_{2}OH\right)}_{2}}\right)\gamma_{{\left(CH_{2}OH\right)}_{2}}=4\left(\frac{\gamma_{{\left(CH_{2}OH\right)}_{2}}^{d}\gamma^{d}}{\gamma_{{\left(CH_{2}OH\right)}_{2}}^{d}+\gamma^{d}}+\frac{\gamma_{{\left(CH_{2}OH\right)}_{2}}^{p}\gamma^{p}}{\gamma_{{\left(CH_{2}OH\right)}_{2}}^{p}+\gamma^{p}}\right)$$

(7) $$\gamma =\gamma^{d}+\gamma^{p}$$

(8) $$\gamma_{H_{2}O}=\gamma_{H_{2}O}^{d}+\gamma_{H_{2}O}^{p}$$

(9) $$\gamma_{{\left(CH_{2}OH\right)}_{2}}=\gamma_{{\left(CH_{2}OH\right)}_{2}}^{d}+\gamma_{{\left(CH_{2}OH\right)}_{2}}^{p}$$

where *γ* is the surface tension, *γ^d^* is the dispersion value, and *γ^p^* is the polarity value. $\theta_{H_{2}O}$ and $\theta_{{\left(CH_{2}OH\right)}_{2}}$ are the contact angles of the substrate with water and the substrate with glycol, respectively. According to the literature [35], $\gamma_{H_{2}O}^{d}=23.9\;\mathrm{dyn}/\mathrm{cm}$, $\gamma_{H_{2}O}^{p}=48.8\;\mathrm{dyn}/\mathrm{cm}$, $\gamma_{{\left(CH_{2}OH\right)}_{2}}^{d}=31.0\;\mathrm{dyn}/\mathrm{cm}$, and $\gamma_{{\left(CH_{2}OH\right)}_{2}}^{p}=17.2\;\mathrm{dyn}/\mathrm{cm}$ in this work.

By combining the dispersion, polarity, and surface tension values of the CR, NR, and MWCNTs (Table 2), it was found that *W_CR–MWCNT_* = 82.3 mN/m and *W_NR–MWCNT_* = 61.5 mN/m. Higher values of *W_AB_* indicate a stronger interaction between the two components. The finding that W*_CR–MWCNT_* > W*_NR–MWCNT_* indicates that there is a stronger interfacial interaction between CR and MWCNTs, which leads to a more efficient transfer of stress from CR to MWCNTs and, thus, a more easily disrupted conductive network when strain occurs.

**Figure 8:**
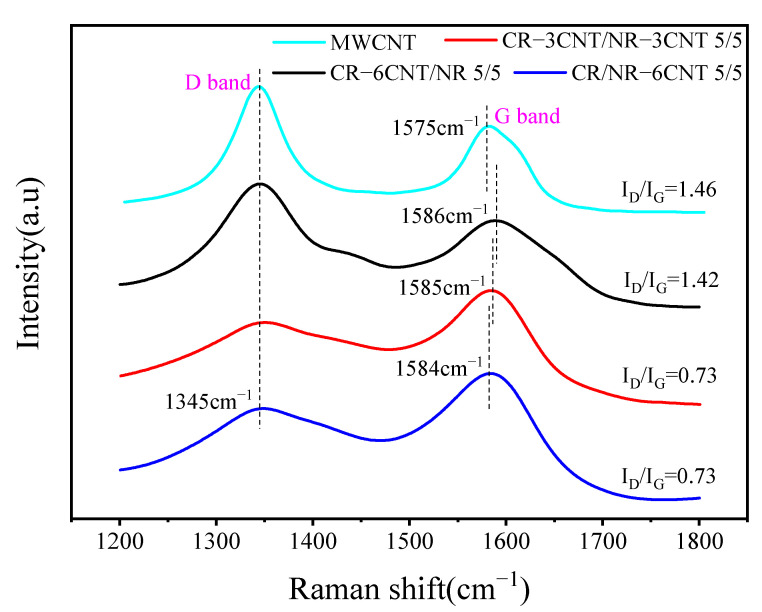
The Raman spectra of MWCNT/CR−NR composites under different blending processes.

Raman analysis was used to further investigate the influence of the blending procedure on the interfacial contact between MWCNTs and the rubber matrix, as shown in Figure 8. There are two strong peaks of MWCNTs at approximately 1345 cm^−1^ and 1575 cm^−1^, which represent the D-peak and G-peak of MWCNTs, respectively. The former is denoted as the D-band induced by structural disorder and flaws and the latter is denoted as the G-band generated by the stretching mode of an sp^2^ atom pair in a carbon ring or long chain. When MWCNTs were blended with rubber, the G-peak of MWCNT/CR–NR composites generated by different blending processes exhibited varying degrees of Raman shifts from 1575 cm^−1^ to 1586 cm^−1^ (CR–6CNT/NR), 1585 cm^−1^ (CR–3CNT/NR–3CNT), and 1584 cm^−1^ (CR/NR–6CNT), while the D-peak did not show a Raman shift. This phenomenon indicates that the MWCNT surface was coated with a rubber film [7]. The ratio of Raman spectral intensities in the D-band and G-band of MWCNT/CR–NR composites *I_D_*/*I_G_* (*I_D_*: D-band intensity, *I_G_*: G-band intensity) can be used to evaluate the interfacial strength of the composites [36]. The *I_D_*/*I_G_* values for the CR–6CNT/NR, CR–3CNT/NR–3CNT, and CR/NR–6CNT composites were 1.42, 0.73, and 0.73, respectively, which are smaller than those of MWCNTs (1.46). This phenomenon may be attributed to the repair of some microscopic defects of MWCNTs during shear, resulting in a simultaneous drop in D-band intensity and an increase in G-band intensity [21]. According to the literature [37], higher *I_D_*/*I_G_* values indicate stronger filler–matrix interactions in the composites, suggesting that the CR–6CNT/NR composites had stronger interfacial interactions than the CR–3CNT/NR–3CNT and CR/NR–6CNT composites.

### 4.2. Morphology of the Conductive Network

**Figure 9:**
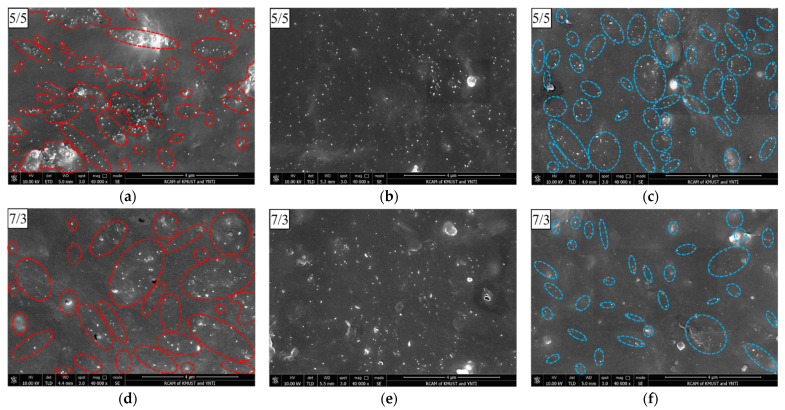
SEM images of MWCNT/CR−NR composites with different blending processes: the red−circled areas are the CR−MWCNT conductive phase, and the blue−circled areas are the NR−MWCNT conductive phase. ((**a**) CR−6CNT/NR 5/5, (**b**) CR−3CNT/NR−3CNT 5/5, (**c**) CR/NR−6CNT 5/5, (**d**) CR−6CNT/NR 7/3, (**e**) CR−3CNT/NR−3CNT 7/3, and (**f**) CR/NR−6CNT 7/3).

For CPCs based on blends, the dispersion of the conductive filler and the morphology of the blend are key factors affecting the conductivity [21]. To confirm the dispersion of MWCNTs under different blending processes, the cross-sectional morphology of MWCNT/CR–NR composites was observed by scanning electron microscopy (SEM). Figure 9 shows the SEM of MWCNT/CR–NR composites with different blending processes (for comparison, the CR/NR blending ratios are 5/5 and 7/3). The small white dots in the figure are all MWCNTs. For CR–6CNT/NR composites (as shown in Figure 9a,d), two distinct regions could be observed: one region contained MWCNTs, and the other region did not contain MWCNTs. Since the ratio of these two regions was consistent with the CR/NR ratio, the region containing MWCNTs was considered the CR phase, and the region without MWCNTs was considered the NR phase. These results occurred because the overall MWCNT/CR blend was continuously penetrated and drained by NR during the blending process of MWCNT/CR blends with NR, thus forming mutually isolated CR–MWCNT conductive phases. For the CR–3CNT/NR–3CNT composites (as shown in Figure 9b,e), the MWCNTs were uniformly dispersed in the matrix because the MWCNTs were located in both CR and NR. For the CR/NR–6CNT composite (as shown in Figure 9c,f), two distinctly different regions were observed, where the MWCNT-containing region corresponded to the NR–MWCNT conductive phase due to the overall MWCNT/NR blend being penetrated and drained by the CR.

Furthermore, because the MWCNT/CR blend was less fluid during rubber compounding than the MWCNT/NR blend, it was easier to form fractured conductive phases, which are more easily penetrated and discharged by NR. The distance between CR–MWCNT conductive phases in CR–6CNT/NR 5/5 composites was farther than the distance between NR–MWCNT conductive phases in CR/NR–6CNT 5/5 composites, resulting in more significant changes in the conductive paths and tunneling distances between the conductive phases in CR–6CNT/NR 5/5 composites under strain. When compared to the CR–3CNT/NR–3CNT 5/5 and CR/NR–6CNT 5/5 composites, the CR–6CNT/NR 5/5 composites forming the conductive phase of CR–MWCNTs had stronger interfacial interactions, and the conductive network was more easily disrupted under strain. Therefore, the CR–6CNT/NR 5/5 composites had the highest strain sensitivity, which was consistent with the above experimental phenomena.

It is due to the formation of the above special conductive network morphology by different blending processes that CR–CNT/NR, CR–CNT/NR–CNT, and CR/NR–CNT composites exhibit different conductive properties (as shown in Figure 5). Among them, CR/NR–CNT composites are more likely to form a tunneling conductive network when the MWCNT content is low due to the closer distance between the conductive phases [38], so CR/NR–CNT composites obtain extremely low percolation thresholds. However, the distance between the conductive phases in the CR–CNT/NR composite is farther, and it is difficult to form conductive paths when the MWCNT content is low. Only when the MWCNT content is high, conductive paths can be formed when the conductive phases are close to each other [39], which is the reason for the high percolation threshold. CR–CNT/NR–CNT composites also have a very low percolation threshold, which is closely related to the good dispersion of conductive fillers in the matrix. It has been reported that evenly dispersed conductive networks have great potential in reducing percolation threshold and forming dense conductive networks [7]. In addition, different blending processes affect the dispersion and network morphology of fillers in the matrix, and then affect the stress transfer and diffusion of composites, resulting in differences in mechanical properties (as shown in Figure 4).

### 4.3. Modeling and Mechanisms

To explore the mechanism of resistance-based strain–sensing behavior during the tensile strain of MWCNT/CR–NR composites produced by three co-blending processes, the following analytical model was developed. According to the tunneling theory model [40], the expression of the total resistance *R* is as follows.

(10) $$R=\left(\frac{M}{N}\right)\left(\frac{8\pi hl}{3\gamma a^{2}e}\right)\exp\left(\gamma l\right)$$

(11) $$\gamma =\frac{4\pi^{2}\sqrt{2m\delta }}{h}$$

where *M* is the number of particles forming a single conducting path, *N* is the total number of conducting paths, *h* is Planck’s constant, *l* is the shortest distance between conducting particles, *a*^2^ is the effective cross–sectional area, *e* is the electron charge, *m* is the electron mass, and *δ* is the height of the potential barrier between neighboring particles.

When uniaxial strain is applied, the resistance will change due to the separation between adjacent conducting particles. The spacing *l* varies linearly with the applied strain *ε* and can be expressed as Equation (12) [41].

(12) $$l=l_{0}\left(1+E\varepsilon \right)$$

where *ε* is the tensile strain of the composite sample, *l*_0_ is the initial distance between adjacent particles, and *E* is a constant.

Due to the high growth rate of resistivity at larger strains, the number of conducting paths is assumed to vary at a higher rate and can therefore be expressed as Equation (13).

(13) $$N=\frac{N_{0}}{\exp\left(\alpha_{1}\varepsilon +\alpha_{2}\varepsilon^{2}+\alpha_{3}\varepsilon^{3}+\alpha_{4}\varepsilon^{4}\right)}$$

where *a*_1_, *a*_2_, *a*_3_, and *a*_4_ are constants.

Substituting Equations (12) and (13) into Equation (10) yields Equation (14).

(14) $$\begin{array}{ll} R & =\frac{8\pi nhl_{0}}{2\gamma N_{0}^{2}a^{2}e^{2}}\left(1+E\varepsilon \right)\exp\left[\gamma l+\left(2\alpha_{1}+\gamma lE\right)\varepsilon +2\alpha_{2}\varepsilon^{2}+2\alpha_{3}\varepsilon^{3}+2\alpha_{4}\varepsilon^{4}\right]\\ & =S\left(1+E\varepsilon \right)\exp\left[N+\left(2\alpha_{1}+NE\right)\varepsilon +2\alpha_{2}\varepsilon^{2}+2\alpha_{3}\varepsilon^{3}+2\alpha_{4}\varepsilon^{4}\right] \end{array}$$

where

(15) $$S=\frac{8\pi nhl_{0}}{2\gamma N_{0}^{2}a^{2}e^{2}}$$

(16) N=γl

Rate of change of resistance ∆*R*/*R*_0_ is as follows:

(17) $$\frac{\Delta R}{R_{0}}=\frac{R}{R_{0}}-1=\left(1+E\varepsilon \right)\exp\left[\left(2\alpha_{1}+NE\right)\varepsilon +2\alpha_{2}\varepsilon^{2}+2\alpha_{3}\varepsilon^{3}+2\alpha_{4}\varepsilon^{4}\right]-1$$

The ∆*R*/*R*_0_-strain curves were fitted by using the theoretical model (Equation (17)), and the fitted parameters are listed in Table 3 (nonlinear curve fitting was used for fitting, and the fitting was carried out until convergence). The variation in the conductive path (CP) and tunneling distance (TD) of the composite under uniaxial tensile strain can be expressed as Equations (18) and (19), respectively [42].

(18) $$y_{CP}=\alpha_{1}\varepsilon +\alpha_{2}\varepsilon^{2}+\alpha_{3}\varepsilon^{3}+\alpha_{4}\varepsilon^{4}$$

(19) $$y_{TD}=E\varepsilon$$

**Figure 10:**
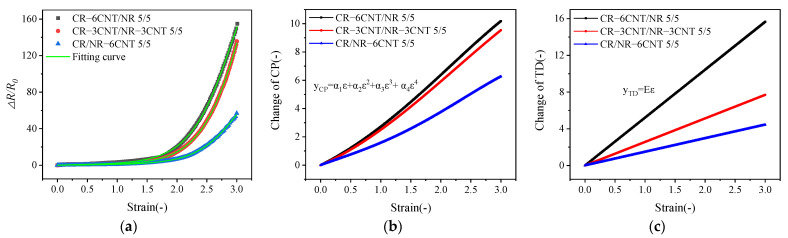
(**a**) Experimental (black, red, and blue dots) and theoretical (green solid line) data for the ∆*R*/*R*_0_−strain curves; (**b**) variation in the conductive path (CP) and (**c**) tunnel penetration distance (TD) versus strain.

As shown in Figure 10a, the tunneling theory model can describe the experimental data well. The variation in CP and TD with strain calculated from the fitted parameters and Equations (18) and (19) are shown in Figure 10b,c, respectively. The increase rates of CP and TD in the CR–6CNT/NR composites were higher than those in the CR–3CNT/NR–3CNT and CR/NR–6CNT composites, indicating that the CR–6CNT/NR composites had greater deformation of the conductive network under strain and thus exhibited a stronger resistance–strain response and higher strain sensitivity, which was consistent with the above experimental results.

### 4.4. Durability and Recyclability

It has been reported that the durability of nano carbon/rubber composites is much higher than that of ordinary rubber due to the addition of nano carbon materials [43,44]. Kong et al. [43] prepared carbon black and graphene modified natural rubber composites and found that when the graphene content was 3 wt.%, the aging and durability of natural rubber were significantly improved, thus enhancing the durability of rubber. Zhang et al. [44] improved the durability of the material by adding modified graphene to the natural rubber matrix to hinder the penetration of oxygen molecules into the rubber. The above research shows that the durability of nano carbon/rubber composites is significantly improved compared with pure rubber materials. MWCNTs have significant advantages in enhancing the durability of rubber materials. Therefore, the durability of composites with different blending processes can be significantly improved with the addition of MWCNTs, which is of great significance for the practical application of MWCNT/CR–NR composites.

Since waste rubber poses a great threat to the ecological environment and human survival [45], the recycling of rubber is of great significance in protecting the environment and saving energy. Therefore, considering the recyclability of materials is crucial to alleviating and solving environmental problems. At present, the common recycling methods of rubber include direct utilization, utilization after crushing into rubber powder, obtaining reclaimed rubber, and fuel utilization. Due to the chemical stability of MWCNTs [46], the incorporation of MWCNTs into rubber will not affect the recyclability of rubber materials. Therefore, it is not necessary to consider that MWCNTs will have a negative impact on the recyclability of rubber materials.

## 5. Conclusions

In this paper, the effects of the blending process on the morphology of the conductive network, interface interaction, and resistance–strain response behavior of MWCNT/CR–NR composites were systematically studied. The results reveal that different blending techniques can provide varied conductive network morphologies and interfacial interactions for controlling resistance–strain response behavior. Based on the results of this study, the following conclusions can be drawn:

(1) Compared with pure CR and NR, CR/NR blends have better tensile strength and elongation at break, and their moduli are between those of pure CR and pure NR.
(2) The composites prepared by different blending processes showed a large strain monitoring range (*ε* = 300%) and good repeatability of the dynamic resistance–strain response. Additionally, the CR–3CNT/NR–3CNT composites have more stable ∆*R*/*R*_0_ amplitude variation compared to the other two blending methods.
(3) Under various blending processes, different conductive network morphologies and interfacial interactions may be formed in the composites, resulting in varied resistance–strain response characteristics. The CR–6CNT/NR composites have the highest strain sensitivity since they contain CR–MWCNTs with a wider conductive phase spacing, stronger interfacial contacts, and significant changes in conductive paths and tunneling lengths under strain.
(4) The resistance–strain response mechanism was investigated by a theoretical model of the tunneling effect. The comparison between the experimental results and the theoretical model shows that the adopted model can well describe and explain the resistance–strain response behavior of the composites.

This study provides a reference to promote the development of MWCNT/CR–NR composites with tunable resistance–strain response behavior in the field of strain sensing and for the selection of excellent co-blending processes. The durability and stability of the dynamic resistance–strain response of MWCNT/CR–NR composites must be explored further. Furthermore, monotonicity as an essential measure for evaluating the quality of a sensing signal and the eradication of the shoulder effect need additional investigation.

## Figures and Tables

**Table 1:** The mixing proportions of MWCNT/CR−NR composites.

Materials	CR−CNT/NR 5/5	CR/NR−CNT 5/5	CR−CNT/NR−CNT 5/5	CR/NR 10/0	CR/NR 7/3	CR/NR 5/5	CR/NR 3/7	CR/NR 0/10
CRL (wt.%)	50	50	50	100	70	50	30	0
NRL (wt.%)	50	50	50	0	30	50	70	100
MWCNT (wt.%)	1, 2, 3, 4, 5, 6	1, 2, 3, 4, 5, 6	1, 2, 3, 4, 5, 6	0	0	0	0	0
MgO (wt.%)	2.5	2.5	2.5	5	3.5	2.5	1.5	0
S (wt.%)	1.5	1.5	1.5	0	0.9	1.5	2.1	3
SA (wt.%)	2	2	2	2	2	2	2	2
ZnO (wt.%)	4	4	4	4	4	4	4	4
4010NA (wt.%)	2	2	2	2	2	2	2	2
NS (wt.%)	1.5	1.5	1.5	1.5	1.5	1.5	1.5	1.5
Blending method	(a)	(b)	(c)	—	—	—	—	—

Method (a): The MWCNTs were first premixed in the CR phase, and then, CR−MWCNTs containing MWCNTs were mixed with NR, and then mixed with other vulcanizing reagents, and finally, the composite was prepared by vulcanization (Figure 2a). Method (b): The MWCNTs were first premixed in the NR phase, and then, NR−MWCNTs containing MWCNTs were mixed with CR and then mixed with other vulcanizing reagents, and finally, the composite was prepared by vulcanization (Figure 2b). Method (c): The MWCNTs were first premixed in the CR and NR phases, separately, and then, CR−MWCNTs containing MWCNTs were mixed with NR−MWCNTs containing MWCNTs, and then mixed with other vulcanizing reagents, and finally, the composite was prepared by vulcanization (Figure 2c). CRL: A wt.%, NRL: B wt.%, A + B = 100.

**Table 2:** The surface tension of MWCNTs, CR, and NR.

Materials	Surface Tension (mN/m)		
	Total (*γ*)	Dispersion Value (*γ^d^*)	Polarity Value (*γ^p^*)
MWCNTs	27.8	17.6	10.2
CR	76.4	14.6	61.8
NR	34.7	17.2	17.5

**Table 3:** The fitting parameters of Equation (17).

Composite	*E*	*N*	*a*_1_	*a*_2_	*a*_3_	*a*_4_	*R* ^2^
CR−6CNT/NR	5.21787	−1.15971	2.12683	0.60131	0.01271	−0.02415	0.99951
CR−3CNT/NR−3CNT	2.56584	−2.12228	1.85823	0.70045	−0.05355	−0.01108	0.99969
CR/NR−6CNT	1.48656	−2.28951	1.51365	−0.16524	0.28264	−0.05449	0.99967

## Data Availability

The data presented in this study are available from the corresponding author upon reasonable request.
